# Supplementary figures and images for: Latitude and longitude as drivers of COVID-19 waves’ behavior in Europe: A time-space perspective of the pandemic
Source: PLoS One. 2023 Sep 15;18(9):e0291618. doi: 10.1371/journal.pone.0291618 (PMC10503727; doi:10.1371/journal.pone.0291618)

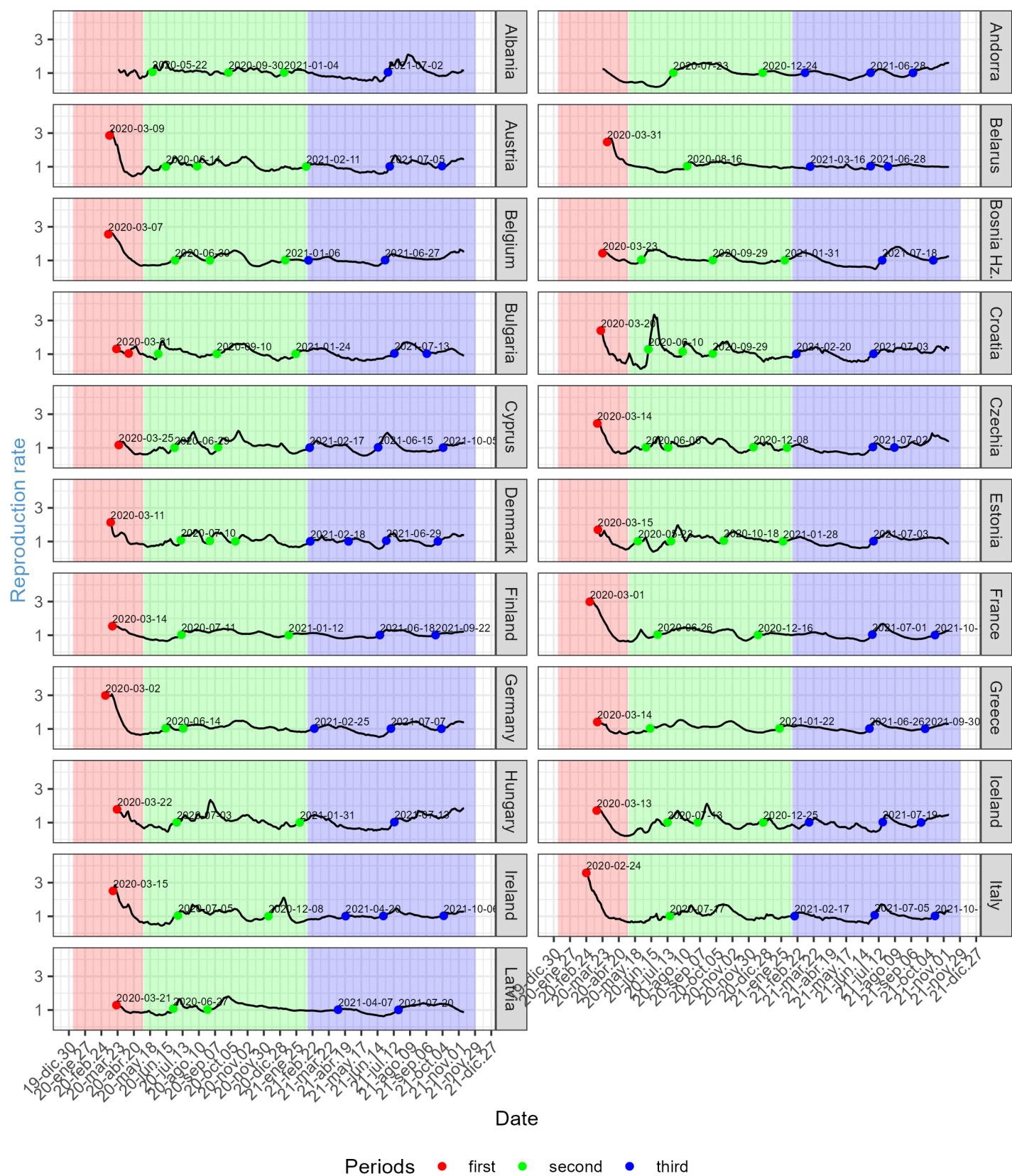

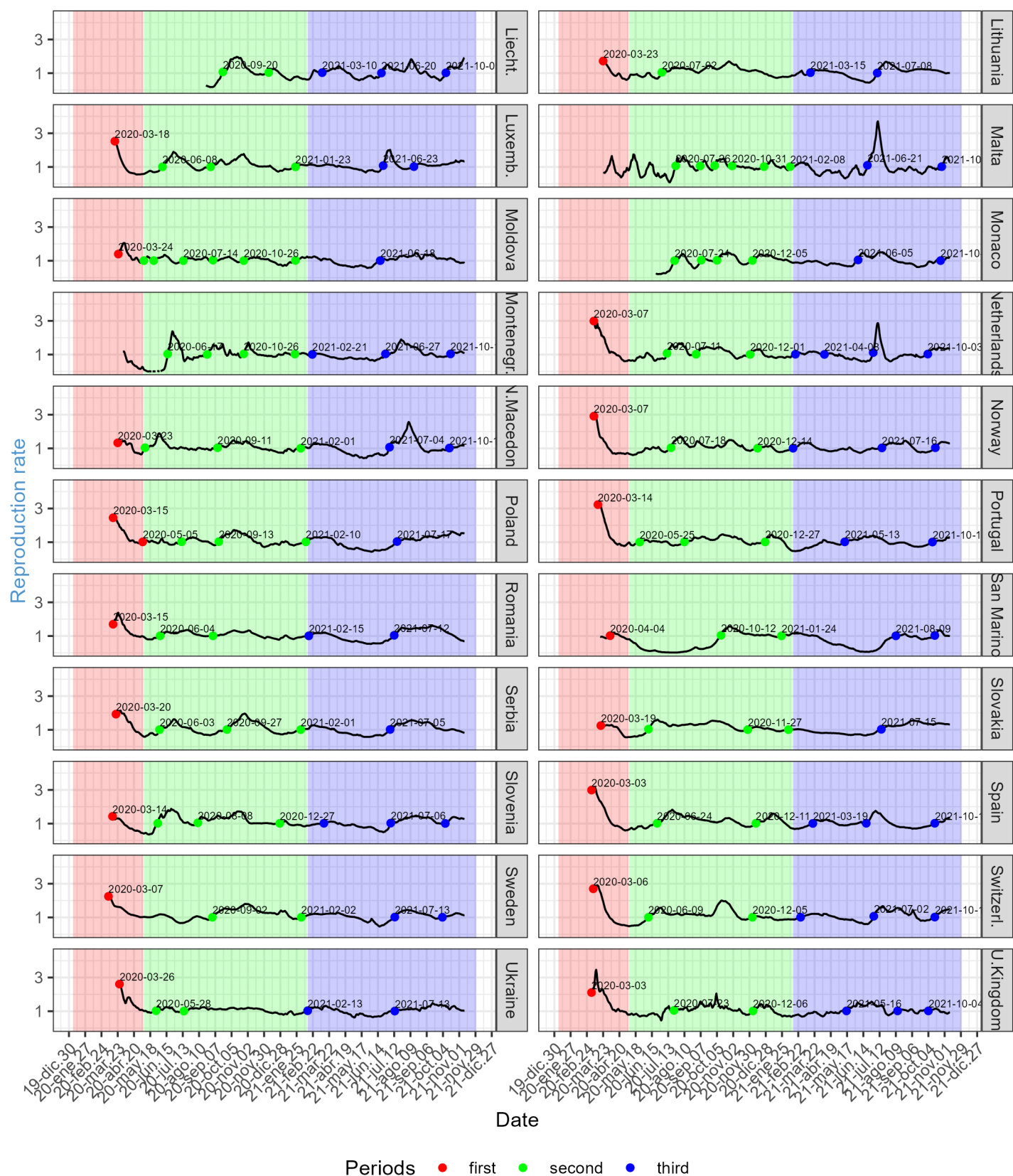

Supplement: S1 Fig — Colored rectangles in the background mark the proposed epidemic periods. Surge dates are colored accordingly to show the periods they were assigned to. (PDF) [file pone.0291618.s001.pdf]
